# The impact of hydroxyethyl starches in cardiac surgery: a meta-analysis

**DOI:** 10.1186/s13054-014-0656-0

**Published:** 2014-12-04

**Authors:** Matthias Jacob, Jean-Luc Fellahi, Daniel Chappell, Andrea Kurz

**Affiliations:** Department of Anesthesiology, Surgical Intensive Care Medicine and Pain Therapy, Harlaching Hospital, Munich Municipal Hospital Group, Munich, Germany; Department of Anesthesiology and Critical Care, Hôpital Cardiovasculaire et Pneumologique Louis Pradel, Hospices Civils de Lyon, 28 avenue du Doyen Lépine, Lyon, Bron Cedex 69677 France; Faculté de Médecine Lyon Est, Université Lyon 1, Lyon, 69008 France; Department of Anesthesiology, University Hospital of Munich, Nussbaumstrasse 20, Munich, 80336 Germany; Department of General Anesthesiology, Cleveland Clinic Main Campus, Mail Code E31, 9500 Euclid Avenue, Cleveland, OH 44195 USA

## Abstract

**Introduction:**

Recent studies in septic patients showed that adverse effects of hydroxyethyl starches (HESs) possibly outweigh their benefits in severely impaired physiological haemostasis. It remains unclear whether this also applies to patient populations that are less vulnerable. In this meta-analysis, we evaluated the impact of various HES generations on safety and efficacy endpoints in patients undergoing cardiac surgery.

**Methods:**

We searched the PubMed, Embase and Cochrane Central Register of Controlled Trials databases for randomised controlled trials (RCTs) in the English or German language comparing the use of HES to any other colloid or crystalloid during open heart surgery.

**Results:**

Blood loss and transfusion requirements were higher for older starches with mean molecular weights more than 200 kDa compared to other volume substitutes. In contrast, this effect was not observed with latest-generation tetrastarches (130/0.4), which performed even better when compared to albumin (blood loss of tetrastarch versus albumin: standardised mean difference (SMD), −0.34; 95% CI, −0.63, −0.05; *P* = 0.02; versus gelatin: SMD, −0.06; 95% CI, −0.20, 0.08; *P* = 0.39; versus crystalloids: SMD, −0.05; 95% CI, −0.20, 0.10; *P* = 0.54). Similar results were found for transfusion needs. Lengths of stay in the intensive care unit or hospital were significantly shorter with tetrastarches compared to gelatin (intensive care unit: SMD, −0.10; 95% CI, −0.15, −0.05; *P* = 0.0002) and crystalloids (hospital: SMD, −0.52; 95% CI, −0.90, −0.14; *P* = 0.007).

**Conclusions:**

In this meta-analysis of RCTs, we could not identify safety issues with tetrastarches compared with other colloid or crystalloid solutions in terms of blood loss, transfusion requirements or hospital length of stay in patients undergoing cardiac surgery. The safety data on coagulation with older starches raise some issues that need to be addressed in future trials.

**Electronic supplementary material:**

The online version of this article (doi:10.1186/s13054-014-0656-0) contains supplementary material, which is available to authorized users.

## Introduction

Hydroxyethyl starches (HESs) have been used as standard solutions for volume replacement therapy for decades. Molecular weight and molar substitution have continuously been adapted to minimise adverse effects such as impairment of blood coagulation or renal function [1].

In 2013, in three reviews and/or meta-analyses, authors have evaluated the effect of HES in different surgical settings. In the first [2], the authors analysed the safety of tetrastarches for mortality, renal function and clinical effects on coagulation in cardiac and non-cardiac surgical patient populations and found no adverse effects. In the second, the authors specifically evaluated renal safety for cardiac and non-cardiac surgical patients and found no adverse effects of HES compared to other fluids [3]. In the third one [4], the authors also did not identify any differences in the incidence of death or acute kidney injury (AKI) in surgical patients. Nevertheless, analysis of a more homogeneous patient population might provide more detailed insights and improve the sensitivity of such analyses. Especially surgical patients with relatively high perioperative blood loss, such as in cardiovascular surgery, might reveal additional safety information.

Shi *et al*. [5] analysed the use of HES in cardiovascular surgery. However, their analysis had two major limitations. First, it contained trials conducted by Boldt *et al*. which later were retracted [6]. Second, it included studies that were not randomised [7] or reported data of unspecified HES. In another analysis, Navickis *et al*. [8] followed a similar strategy, but did not distinguish between different generations of HES.

In the present meta-analysis, we reevaluate the use of HES in cardiac surgery, separately analysing potential negative effects of different generations. Cardiac surgery seems to be an ideal setting for safety evaluation due to the high need for volume replacement that should best reveal adverse effects.

## Methods

We searched various databases for randomised controlled clinical trials in open heart surgery in the English or German language in which researchers compared HES to any other fluid. Trials comparing HES to fresh frozen plasma (FFP) were excluded, as FFPs are no longer recommended. There was no time restriction, and all stages of publication were eligible. The manuscript was prepared according to the preferred reporting items for systematic reviews and meta-analyses (PRISMA) statement [9]. Because the meta-analysis was based on published study data, approval by an ethics committee was waived. As well, no patient consent was needed.

### Literature search

Studies were identified by searching the PubMed (1946 to July 2013), Embase (1974 to July 2013) and Cochrane Central Register of Controlled Trials (1993 to July 2013) databases. Our search strategy can be found in Additional file 1. Any systematic reviews were checked for further suitable publications.

### Study selection

We removed duplicates from all hits, and two independent reviewers identified studies meeting the eligibility criteria based on the title and/or abstract. Any disagreements were resolved by consulting the full text of the article, discussion between the reviewers or consultation with a third reviewer.

### Data acquisition

All data were collected on predesigned data extraction sheets by two experienced reviewers. In cases of any discrepancies, a third reviewer was consulted. The safety, efficacy and HES data listed below were extracted from the included publications.

1. Safety:
  - Volume of total blood loss within 24 hours after surgery
  - Frequency of blood transfusions within 24 hours after surgery
  - Frequency of reoperations
  - Frequency of AKI during hospitalisation
  - In-hospital mortality
  - Length of stay in intensive care unit (ICU)
  - Length of stay in hospital

2. Efficacy: combined volume of colloids and crystalloids

3. HESs were classified according to their molar substitution:
  - Tetrastarch 0.4 (molecular weight (MW), 130 kDa),
  - Pentastarch 0.45 (MW, 264 kDa),
  - Pentastarch 0.5 (MW, 120, 200 or 250 kDa),
  - Hetastarch 0.7 (MW, 400 or 450 kDa).

Each of the HES groups was compared to albumin, crystalloids and gelatin.

#### Total blood loss

The total blood loss endpoint was defined as blood loss from the start of the operation until 24 hours after surgery. If data were not available for the complete time frame, the largest available time span was selected for analysis. In some studies, blood loss data were not available for the complete time interval, but were obtained for several adjacent partial intervals. These intervals were combined by adding the mean blood loss for each of the partial intervals and by calculating the standard deviation of the sum of these means, assuming a negative correlation of about 0.5 between the intervals. If calculated blood loss was reported, we preferentially chose this parameter because it is more accurate, as it accounts for non-exteriorised blood losses.

#### Blood transfusions

The proportion of patients receiving blood transfusions (packed red blood cells) intraoperatively and up to 24 hours after surgery was evaluated. Because the number of patients receiving blood transfusions often was presented for separate time intervals, the total number of patients was estimated by determining the harmonic mean of all possible outcomes rounded to the nearest integer. The percentages of patients receiving blood transfusions were used for calculation of the number of patients receiving blood transfusions rounded to the nearest integer. Publications that presented only the number of patients receiving allogeneic blood products were included in the meta-analysis using the number of patients who received any allogeneic blood product as a substitute for the number of patients who received packed red blood cells.

#### Volume infused

The endpoint “total combined volume of colloids and crystalloids” was defined as the total amount of colloids (that is, HES, albumin, and gelatin) and crystalloids administered from the start of the operation until 24 hours afterwards. If data were not available for the entire time interval, the largest available interval was selected for analysis. Volumes added via cardiopulmonary bypass (CPB) were considered because they add to intraoperative volume expansion. In some cases, volumes had to be combined because different types of colloids and crystalloids were infused or because fluid volumes were presented not for the complete time interval, but for several adjacent intervals. In these instances, fluid volumes were combined by adding the mean volume for each of the fluids in each of the intervals and calculating the standard deviation of the sum of these means, assuming a negative correlation of 0.5 between the fluid volumes in the partial intervals.

#### Length of stay in ICU and in hospital

“Length of stay in ICU” and “length of stay in hospital” were defined as the number of days spent in the ICU or hospital after the end of surgery. If data for length of stay were not presented as mean ± standard deviation, it was assumed that the distribution of data was symmetrical and approximately normal. Thus, median values were used with the interquartile ranges divided by 1.35 as an estimate of standard deviation.

#### Acute kidney injury, mortality and need for reoperation

The number and proportion of patients who needed reoperation were considered as mentioned by the study authors, regardless of any specific time window. The same approach was applied for AKI and mortality.

### Statistical analysis

For effect size estimation for continuous endpoints, we used standardised mean difference (SMD); for binary endpoints, we used risk ratios (RRs). Fixed-effects models were applied to derive common point estimates and associated 95% confidence intervals (CIs). The common effect estimate was calculated as a weighted average of the effects estimated in the individual studies. For continuous endpoints, the inverse-variance approach was used; for binary endpoints, the Mantel-Haenszel approach was applied [10]. A zero-cell correction was performed for studies. If cells with the value 0 occurred, 0.5 was added to all cells of the respective contingency table, except for cases where no events were observed in both treatment groups. A heterogeneity test was applied for each meta-analysis. In case of significant results (*P* ≤0.05), possible causes were analysed and discussed. In particular, if all study-specific results pointed consistently in the same direction, valid interpretation of the common effect estimate sometimes still was possible. Statistical analyses were performed using the RevMan 5.2 software package (The Nordic Cochrane Centre, Copenhagen, The Cochrane Collaboration, 2013).

## Results

**Figure 1:**
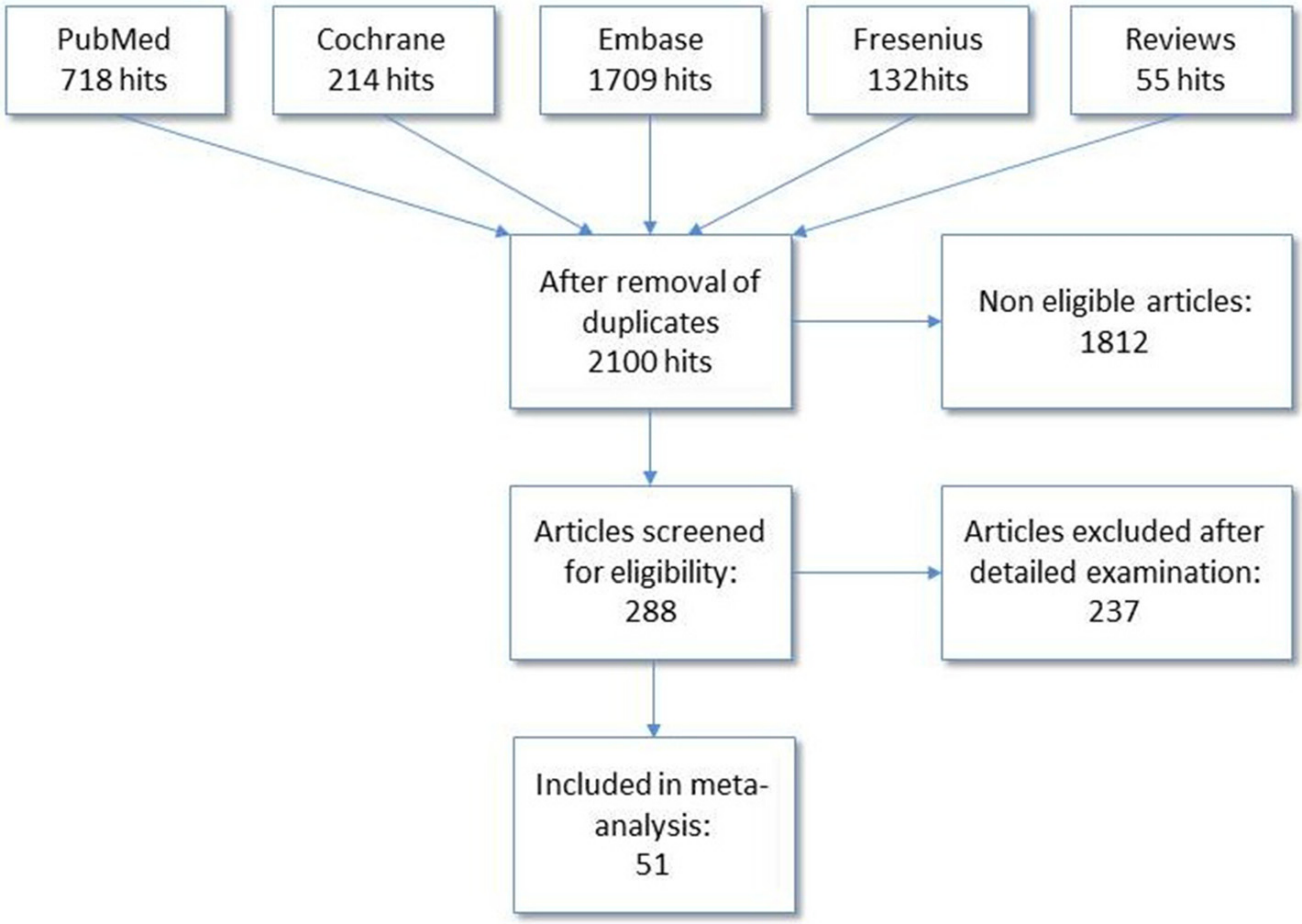
**Overview of study selection.**

For overview of study selection, please refer to Figure 1.

The literature search yielded 2,100 hits, from among which 288 were retrieved for detailed evaluation. Two hundred thirty-seven of these studies were excluded for the following reasons:

- Fifty-five were not prospective, randomised studies in cardiac surgery patients published in the English or German language.
- Fifty-two were not original research articles; that is, they were abstracts, letters or comments.
- Thirty-three were published in languages other than English or German.
- Seven did not have a full text available.
- Twenty-three were reviews or meta-analyses.
- Seventeen used no comparator or a comparator not prespecified by us.
- Nineteen were duplicates or publications of the same study providing no additional data.
- Twelve did not use HES as a study medication.
- Nine papers were retracted.
- Ten did not provide endpoints evaluated by our analysis.

Thus, we ultimately included in our meta-analysis 51 publications describing 49 clinical studies composed of an aggregate of 3,439 patients. Of these 49 studies, 30 [11-42] were unblinded, 10 [43-52] were partly blinded and 9 were completely blinded [53-61]. The duration of follow-up covered a wide range, from 2 hours to 30 days. Patients received on-pump coronary artery bypass grafting in 25 studies, off-pump coronary artery bypass grafting in 7 studies and mixed on-pump cardiac surgery in 15 studies. In four studies, the cardiac interventions performed were not specified.

In two studies, the researchers reported additional data about one of these trials. A total of 2,114 patients were included in studies comparing tetrastarches to albumin (*n* = 185), gelatin (*n* = 888), crystalloids (*n* = 679) and other starches (*n* = 342). For pentastarches and hetastarches, a total of 854 patients were included. Because more than two substances were compared to each other in some trials, the number of patients for single comparisons does not always match the total number of patients.

### Safety evaluation

#### Total blood loss

Total blood loss was reported in 17 studies in which starches were compared to albumin, 17 to crystalloids and 14 to gelatin.

Niemi *et al*. [32] and Schramko *et al*. [35] (both publications of the same study) seem to have mixed up the results for HES treatment regimens. Therefore, it is not clear which data are correct; we chose the more severe blood loss of 895 ml for both starches in the meta-analysis. Furthermore, Hanart *et al*. [43] reported total blood loss in two ways; we used calculated blood loss.

**Figure 2:**
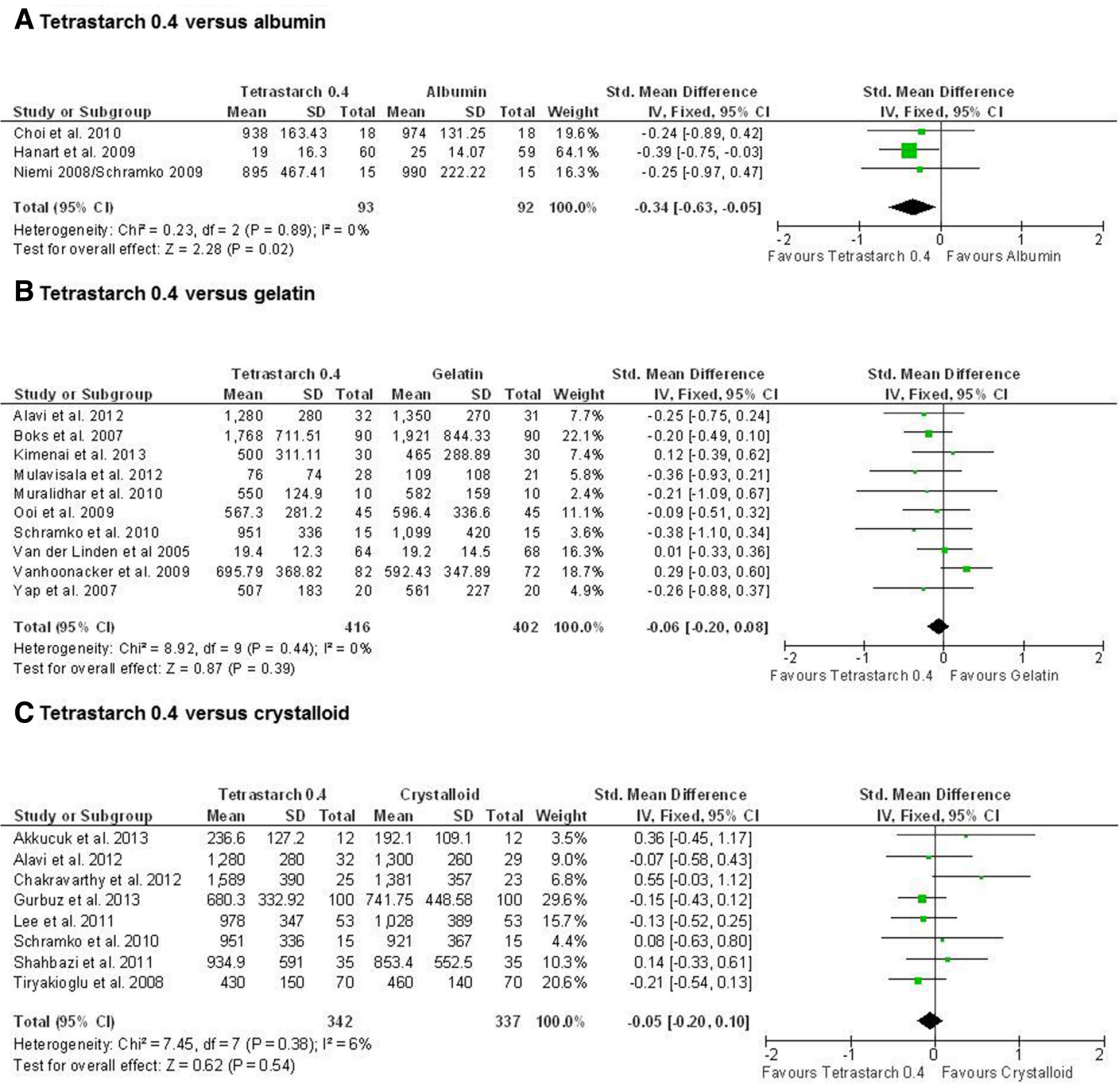
**Blood loss with tetrastarch compared to albumin, gelatin or crystalloids.** Units of blood loss were expressed in millilitres (ml), except for Hanart *et al*. [43] and Van der Linden *et al*. [52], where the units were millilitres per kilogram body weight, and Lee *et al*. [47], where no unit was indicated. The standardised mean difference (Std. mean difference) of the mean for the tetrastarch groups minus the mean for the albumin **(A)**, gelatin **(B)** and crystalloid **(C)** groups was used as effect size. Fixed-effects models were applied to calculate a common effect estimate using the inverse-variance method (IV). SD, standard deviation; CI, Confidence interval.

**Figure 3:**
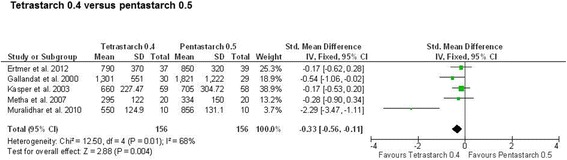
**Blood loss with tetrastarch compared to pentastarch.** Units of blood loss were millilitres (ml). The standardised mean difference (Std. mean difference) of the mean for the tetrastarch groups minus the mean for the pentastarch 0.5 group was used as effect size. A fixed-effects model was applied to calculate a common effect estimate using the inverse-variance method (IV). SD, Standard deviation; CI, Confidence interval.

For comparison of tetrastarch to albumin, three studies reported lower blood loss with tetrastarch (Figure 2) (SMD, −0.34; 95% CI, −0.63, −0.05; *P* = 0.02). For crystalloids and gelatins, no significant difference compared to tetrastarches was found (tetrastarch vs. gelatin: SMD, −0.06; 95% CI −0.20, 0.08; p = 0.39; tetrastarch vs. crystalloids: SMD, −0.05; 95% CI −0.20, 0.10; *P* = 0.54). In contrast, for penta- or hetastarches, all analyses showed at least a trend in favour of the comparator or even significantly larger blood loss with the older starches (Additional files 2 and 3). This difference between HES generations was also confirmed by studies comparing starches with each other [27,30,46,54,55], with tetrastarch being associated with significantly lower blood loss compared to pentastarch (SMD, −0.33; 95% CI, −0.56, −0.11; *P* = 0.004) (Figure 3). Significant heterogeneity amongst studies was found for this comparison, but a sensitivity analysis excluding the Muralidhar *et al*. [30] study confirmed the result for tetrastarches (SMD, −0.26; 95% CI, −0.49, −0.03; *P* <0.05).

#### Need for transfusions

The need for transfusion was reported in nine studies with HES vs. albumin, in eleven with HES vs. crystalloids and in nine with HES vs. gelatin. No significant heterogeneity was found for these comparisons.

**Figure 4:**
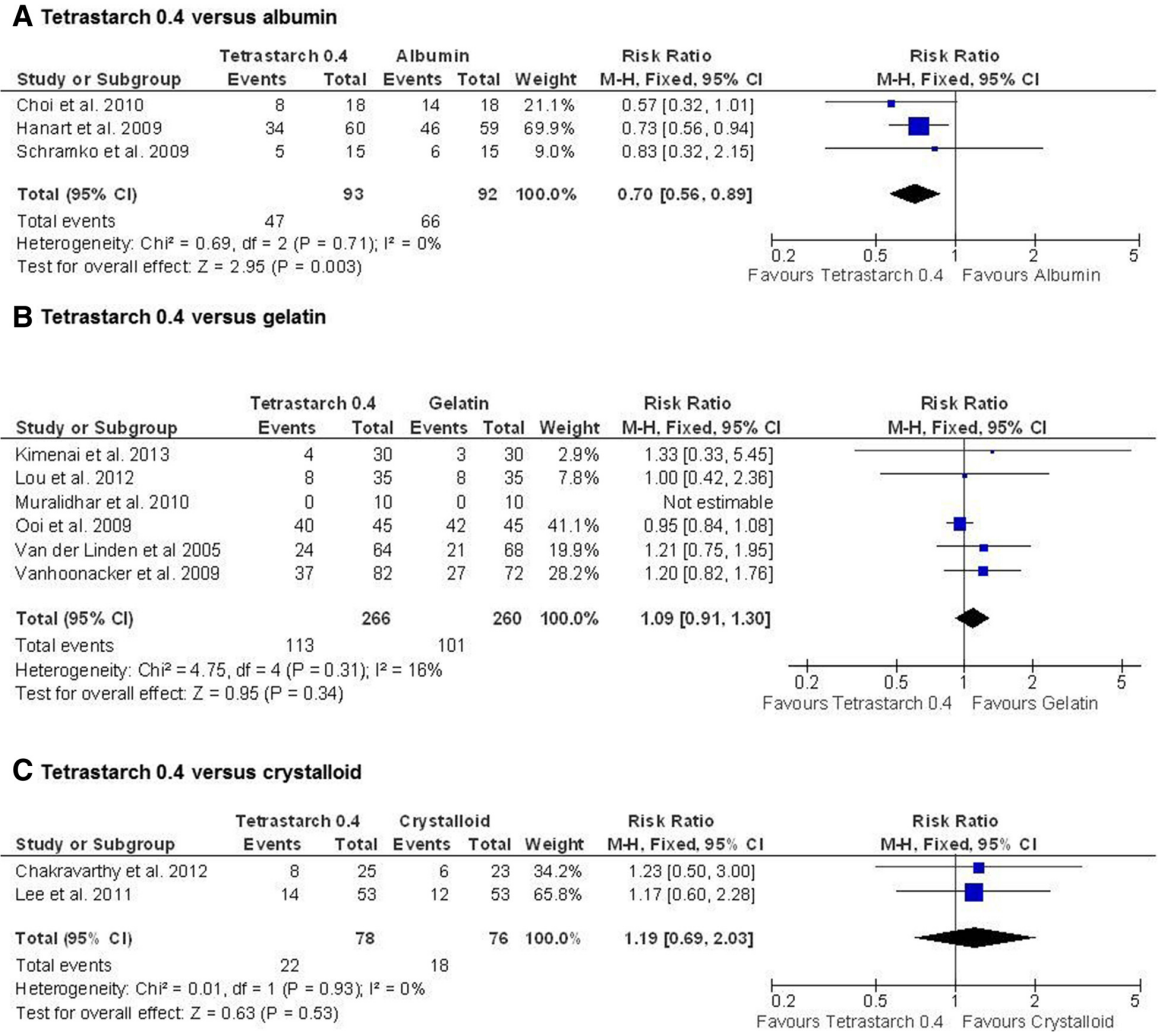
**Transfusion requirements after tetrastarch compared to albumin, gelatin or crystalloids.** The risk ratio was used as effect size (transfusion risk for the hydroxyethyl starch groups divided by transfusion risk for the albumin **(A)**, gelatin **(B)** and crystalloid **(C)** groups). Fixed-effects models were applied to calculate a common effect estimate using the Mantel-Haenszel (M-H) approach. CI, confidence interval.

As for blood loss, Niemi *et al*. [32] and Schramko *et al*. [35] (both publications of the same study) did not report the same results for the HES treatment regimens. Because the study medication was applied postoperatively and only the publication by Schramko *et al*. presents numbers of patients receiving transfusions after surgery, only the data reported by Schramko *et al*. were included in the meta-analysis. Patients receiving tetrastarch 0.4 needed significantly less blood transfused for cardiovascular surgery compared to patients receiving albumin (RR, 0.70; 95% CI, 0.56; 0.89) (Figure 4).

No differences were found for tetrastarch compared to crystalloids or gelatin (tetrastarch vs. gelatin: RR, 1.09; 95% CI, 0.91, 1.30; *P* = 0.34; tetrastarch vs. crystalloids: RR, 1.19; 95% CI, 0.69, 2.03; *P* = 0.53) (Figure 4): For crystalloids, only two of eight publications could be used for meta-analysis of blood transfusion risk, and they showed no significant difference in transfusion needs between both fluids (RR, 1.19; 95% CI, 0.69, 2.03; *P* = 0.53). No transfusion events were reported in the remaining six studies, but different data were reported about transfusions. The mean volume of packed red blood cells was lower for tetrastarch in one study [11], but not the number of patients who received them. In another study, the mean number of packed red blood cells was lower with tetrastarch [19]. In one study [42], the researchers reported that equal volumes of red blood cells were required, without regard to whether volume expansion was performed with tetrastarch or crystalloids. In two studies, the investigators reported that more packed red blood cells were transfused with tetrastarch [51,53].

For older starches, sparse data were available. Pentastarch was associated with higher transfusion needs compared to albumin (RR, 1.77; 95% CI, 1.12, 2.79; *P* = 0.01), gelatin (RR, 2.86; 95% CI, 1.39, 5.88; *P* = 0.004) and crystalloids (RR, 1.35; 95% CI, 0.93, 1.97; *P* = 0.11) (Additional file 4), but the number of studies included in these comparisons was low.

In four studies, researchers reported higher transfusion needs for hetastarch compared to albumin (RR, 1.48; 95% CI, 1.04, 2.10; *P* = 0.03). In only two studies were data reported related to blood transfusions with hetastarch compared to crystalloids. There was no difference between the two treatments, but available data were sparse [34,57].

#### Acute kidney injury, mortality and need for reoperation

For the outcome parameters AKI, mortality and need for reoperation, only very few events were reported: for example, only 29 events for AKI in 1,538 cardiac surgical patients for all HES cases. The overall incidence of mortality was 5 deaths among 745 patients (0.7%) for all HES cases and 8 deaths among 793 patients (1.0%) for all comparators. Although there was no trend towards a difference between starches and comparators, no reliable analysis for separate HES generations compared to albumin, gelatin or crystalloids was possible. However, the need for reoperations in a pooled analysis of each starch generation compared to all comparators showed that it was significantly greater with hetastarches (RR, 2.85; 95% CI, 1.27, 6.42; *P* = 0.01), whereas it did not differ between tetrastarches (RR, 1.20; 95% CI, 0.39, 3.69; *P* = 0.75) or pentastarches (RR, 1.64; 95% CI, 0.51, 5.24; *P* = 0.41) and other volume replacements.

#### Length of stay in ICU and hospital

**Figure 5:**
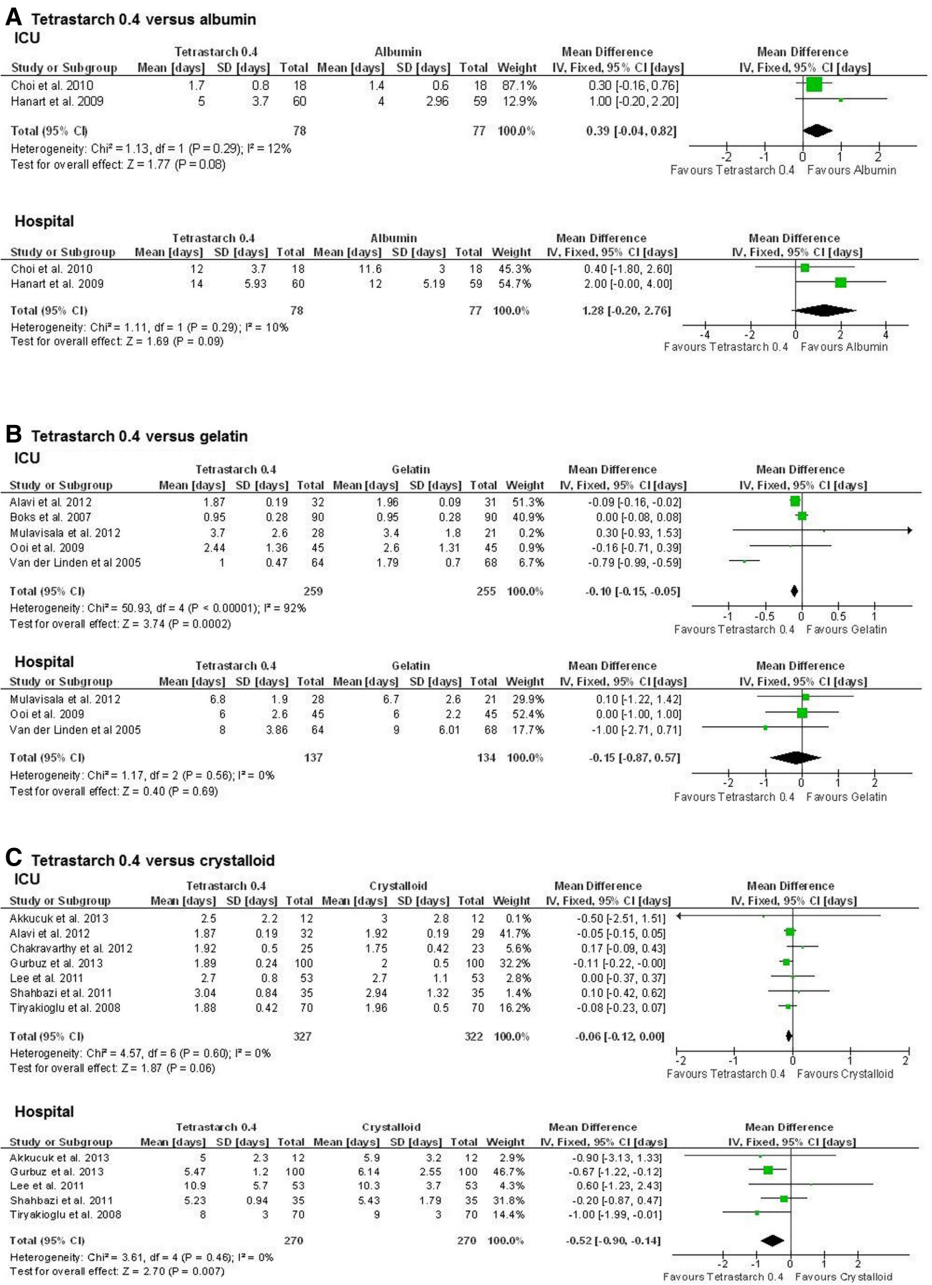
**Length of stay in the ICU or the hospital after tetrastarch compared to albumin, gelatin or crystalloids.** The standardised mean difference (Std. mean difference) of the mean for the hydroxyethyl starch groups minus the mean for the albumin **(A)**, gelatin **(B)** and crystalloid **(C)** groups was used as effect size. A fixed-effects model was applied to calculate a common effect estimate using the inverse-variance method (IV). ICU, Intensive care unit; SD, Standard deviation; CI confidence interval.

Compared to albumin, there was a trend towards longer ICU and hospital lengths of stay for tetrastarches which was not statistically significant (ICU: SMD, 0.39; 95% CI, −0.04, 0.82; *P* = 0.08; hospital: SMD, 1.28; 95% CI −0.20, 2.76; *P* = 0.09). In contrast, tetrastarch seemed to have been associated with shorter ICU length of stay and similar hospital length of stay compared to gelatin (ICU: SMD, −0.10; 95% CI, −0.15, −0.05; *P* = 0.0002; hospital: SMD, −0.15; 95% CI, −0.87, 0.52; *P* = 0.69). Compared to crystalloids tetrastarches were associated with significantly shorter lengths of stay in the ICU (SMD, −0.06; 95% CI, −0.12, 0.00; *P* = 0.06) and in the hospital (SMD, −0.52; 95% CI, −0.90, −0.14; *P* = 0.007) (Figure 5). However, again, no definite conclusion is possible because of the low number of studies.

Similarly, for penta- and hetastarches, the number of included studies is too low to allow definitive conclusions. These comparisons can be found in Additional files 5 and 6.

### Efficacy evaluation

#### Volume infused

**Figure 6:**
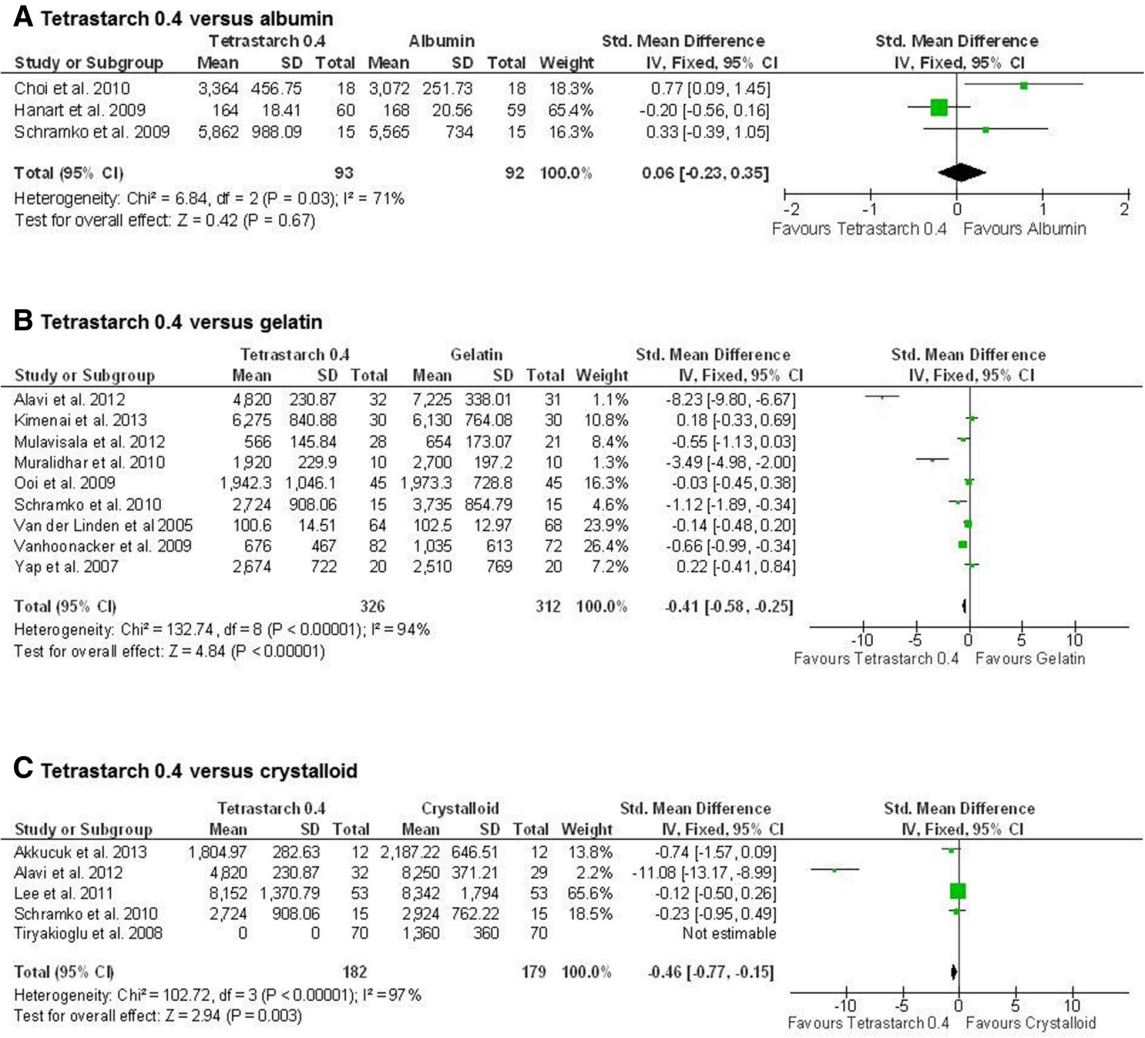
**Efficacy of tetrastarch compared to albumin, gelatin or crystalloids as judged by total volume infusion.** Units of total combined volume of colloids and crystalloids were expressed in millilitres (ml), except for Hanart *et al*. [43] and Van der Linden *et al*. [38,52], where units were expressed as millilitres per kilogram body weight (ml/kg). **(A)** Albumin. **(B)** Gelatin. **(C)** Crystalloid. The standardised mean difference (Std. mean difference) of the mean for the hydroxyethyl starch group minus the mean for the albumin group was used as effect size. Fixed-effects models were applied to calculate a common effect estimate using the inverse variance method (IV). SD, standard deviation; CI, confidence interval.

The need for fluids was assessed in 8, 12 and 14 studies comparing HES to crystalloids, gelatin and albumin, respectively. Our analysis shows no difference between tetrastarch and albumin (SMD, 0.06; 95% CI, −0.23, 0.35; *P* = 0.67). Significant heterogeneity was indicated for this comparison, most likely due to the low number of studies. In contrast, a significantly lower volumetric need was found when we compared tetrastarches to gelatin (SMD, −0.41; 95% CI, −0.58, −0.25; *P* <0.00001) and crystalloids (SMD, −0.46; 95% CI, −0.77, −0.15; *P* = 0.003) (Figure 6). Significant heterogeneity was also found for these analyses. For tetrastarch vs. gelatin, the study results reported by Muralidhar *et al*. [30] and Alavi *et al*. [53] appear most relevant for heterogeneity, but these results pointed in the same direction as those from most other trials. Pentastarch 0.45 was as effective as albumin (SMD, 0.18; 95% CI, −0.17, 0.54; *P* = 0.31) and gelatin (SMD, −0.22; 95% CI, −0.53, 0.10; *P* = 0.18), whereas pentastarch 0.5 was inferior to albumin (SMD, 0.57; 95% CI, 0.10, 1.04; *P* = 0.02) (Additional file 6). For hetastarch, only sufficient data for a comparison to albumin were available, indicating similar efficacy of both fluids (SMD, 0.08; 95% CI, −0.13, 0.28; *P* = 0.47) (Additional file 6).

## Discussion

Our meta-analysis shows no evidence for a higher risk of bleeding, blood transfusion or reoperation associated with the third generation of HESs in patients undergoing cardiac surgery. For blood loss and transfusion needs, we found a trend amongst starches (that is, a reduction of adverse effects) with third-generation starches. Tetrastarch was superior to human albumin in terms of blood loss and transfusion requirements. The efficacy of tetrastarches, as judged by the amount needed for haemodynamic stabilisation, was superior to crystalloids and gelatin. Length of stay in the ICU or in the hospital could be shorter with tetrastarches as compared to gelatins or crystalloids. In contrast, using starches with a higher degree of molar substitution was associated with adverse outcomes. Thus, our analysis indicates that the development of newer-generation starches might have improved the safety profile of HESs substantially over time.

### Safety evaluation

Because of large volumes of priming solution and volume replacement, blood loss and transfusion requirements are especially relevant in cardiac surgery. A central safety aspect of starches is their effect on coagulation. Generations with a higher degree of molar substitution (0.5 or higher) have been shown to impair coagulation [62,63]. The results of several studies comparing tetrastarches with a molar substitution of 0.4 to other generations suggest a smaller effect on coagulation with tetrastarches [1,64]. Our meta-analysis shows a reduction of blood loss and need for transfusion with decreasing molar substitution from heta- and penta- to tetrastarches. When we compared blood loss and transfusion requirements with tetrastarches, we found no significant difference compared to crystalloids and gelatin. Furthermore, blood loss and transfusion requirements with tetrastarches were significantly lower compared to albumin. However, this result was based on only 3 studies with 185 patients and thus should be viewed as preliminary. In contrast, penta- and hetastarches were inferior in terms of blood loss and transfusion requirements when compared to other volume replacements in our analysis.

Other safety parameters we analysed were overall mortality, the incidence of AKI and the need for reoperations, which might also indicate bleeding events. Firm conclusions could not be drawn for AKI and mortality, owing to a very low number of reported events. For AKI, there is an overall trend towards providing specific definitions only in the later studies, which mainly relied on creatinine values (for example, peak creatinine value at least 50% above baseline) and need for renal replacement therapy. In a recently published trial by Van der Linden *et al*. [65], tetrastarch was compared with albumin in paediatric cardiac surgery. They assessed safety parameters until 28 days after surgery and monitored highly sensitive markers of renal function, but they could not detect significant differences between groups. With regard to the need for reoperations, a pooled analysis of all starches showed no difference compared to other volume substitutes, which confirms the data about blood loss and transfusions.

With regard to length of stay in the ICU or in the hospital, tetrastarches seem to be superior to crystalloids and gelatin. Albumin might offer advantages in terms of length of stay compared to tetrastarches. As length of stay in the hospital is a parameter that is especially prone to non-medical confounders, such as availability of secondary care or weekend discharges, the validity of this endpoint may be lower than that for other safety endpoints, given the low number of studies and patients available for this comparison.

### Efficacy evaluation

Generally, volume used for fluid therapy is a tricky parameter in assessment of the efficacy of therapy, as this need is judged by physicians on the basis of different parameters, such as volumetric parameters or cardiac preload. Yet, it is the only parameter consistently reported in studies on volume therapy. In addition, combining study results might be more reliable than evaluating single studies, as individual deviations in judgement about the need for fluid therapy might regress towards the mean. The volume effect of colloids has been discussed extensively and also controversially [66,67]. Our analysis of the total amount of fluid replacement drugs per case indicates that volume therapy with tetrastarches required significantly less volume than with crystalloids. Compared to gelatin, tetrastarch use also led to the infusion of significantly lesser total amounts, supporting the notion that they might have a greater volume effect [68,69]. However, significant heterogeneity was found amongst the study populations. Therefore, this result needs further confirmation. More studies would be necessary to explore potential sources of heterogeneity. Compared to human albumin, second-generation HES with a molar substitution of 0.5 (pentastarches) seem to be less efficient, which was not the case for tetrastarches. This finding is consistent with previous studies in which researchers reported similar volumetric effects for albumin and tetrastarches [68-71]. However, to draw reliable conclusions about intravascular volumetric effects from total infused amounts over time guided by often insufficient routine surrogates (for example, blood pressure, heart rate) is problematic. Beyond that, significant heterogeneity was found amongst the study populations. Therefore, this result needs to be confirmed. More studies using adequate targets of fluid therapy would be necessary to explore and exclude potential sources of heterogeneity and to focus on what we actually want to know when talking about intravascular persistence.

### Limitations

Dosing of colloids was markedly different amongst studies, which were conducted worldwide, possibly resulting in different treatment regimens that might result in relevant heterogeneity. This might affect the results, especially owing to the low number of studies for some of the comparisons. However, it increases the external validity of our findings. Publication bias existed, as described previously [5]. For comparison of efficacy, no data from extended monitoring devices were available beyond the weak surrogate parameter of the volume of infused fluids.

Borderline sufficient data were available for some of our analyses. Also, statistical significance for our comparisons does not automatically imply clinical relevance. However, there is an overall trend that starches have been improved over time with tetrastarches providing the most reliable data.

## Conclusions

Recent studies in septic patients suggest that adverse effects of HESs might outweigh the benefits in these patients [72,73]. On the other hand, it has to be taken into account that the pathophysiology of these patients differs fundamentally from surgical patient populations not primarily presenting with a capillary leak negatively affecting colloidal intravascular persistence [74]. As the safety debate for surgical patients is ongoing, an evaluation of HESs for specific patient populations seems to be mandatory.

We conclude that tetrastarches are improved compared to older starches in regards to blood loss or need for transfusions. On the basis of the available data, tetrastarches seem to be efficient and safe volume substitutes which can be recommended for cardiac surgery.

## Key messages

- Tetrastarches are improved in regards to blood loss or need for transfusions compared to older starches.
- We found no safety issues with tetrastarches in terms of blood loss, transfusion requirements or hospital length of stay in cardiac surgery.
- Volume therapy with tetrastarches required significantly less volume than with crystalloids and gelatin.

## Additional files

Additional file 1
**Target terms for literature search.**
Additional file 2
**Blood loss with pentastarch compared to albumin, gelatin or crystalloids.** Units of blood loss were millilitres (ml), except for Van der Linden *et al*. [52], where units were millilitres per kilogram body weight. The standardized mean difference of the mean for the pentastarch groups minus the mean for the albumin, gelatin and crystalloid groups was used as effect size. Fixed-effect models were applied to calculate a common effect estimate using the inverse variance method. Tigchelaar *et al*. [37] only report mean blood loss without presenting standard deviation (indicated by a ‘0’ in this figure). SD, Standard deviation; Std. mean difference, Standardized mean difference; IV, Inverse variance method; CI, Confidence interval. Additional file 3
**Blood loss with hetastarch compared to albumin or crystalloids.** Units of blood loss were millilitres (ml), except for Brutocao *et al*. [14], where units were millilitres per kilogram body weight (ml/kg), and Palanzo *et al*. [41], where no unit was indicated. The standardized mean difference of the mean for the hetastarch groups minus the mean for the albumin and crystalloid groups was used as effect size. Fixed-effects models were applied to calculate a common effect estimate using the inverse variance method. SD, Standard deviation; Std. mean difference, Standardized mean difference; IV, Inverse variance method; CI, Confidence interval. Additional file 4
**Transfusion requirements after penta- or hetastarch compared to albumin, gelatin, or crystalloids.** The risk ratio was used as effect size (transfusion risk for the penta- or hetastarch groups divided by transfusion risk for the albumin, gelatin and crystalloid groups). Fixed effect models were applied to calculate a common effect estimate using the Mantel-Haenszel approach. M-H, Mantel-Haenszel approach; CI, Confidence interval. Additional file 5
**Length of stay in ICU or hospital after pentastarches compared to albumin and crystalloids.** The standardized mean difference of the mean for the pentastarch group minus the mean for the albumin group was used as effect size. A fixed-effects model was applied to calculate a common effect estimate using the inverse variance method. SD, Standard deviation; Std. mean difference, Standardized mean difference; IV, Inverse variance method; CI, Confidence interval. Additional file 6
**Length of stay in ICU or hospital after hetastarches compared to albumin and crystalloids.** The standardized mean difference of the mean for the pentastarch group minus the mean for the albumin group was used as effect size. A fixed-effect model was applied to calculate a common effect estimate using the inverse variance method. SD, Standard deviation; Std. mean difference, Standardized mean difference; IV, Inverse variance method; CI, Confidence interval.

## Abbreviations

AKI: Acute kidney injury
CI: Confidence interval
CPB: Cardiopulmonary bypass
EMA: European Medicines Agency
FFP: Fresh frozen plasma
HES: Hydroxyethyl starch
ICU: Intensive care unit
MW: Molecular weight
PRAC: Pharmacovigilance Risk Assessment Committee
PRISMA: Preferred reporting items for systematic reviews and meta-analyses RCT, Randomised controlled trial
RR: Risk ratio
SMD: Standardised mean difference
