# Supplementary material for: The impact of hydroxyethyl starches in cardiac surgery: a meta-analysis
Source: Crit Care. 2014 Dec 4;18(6):656. doi: 10.1186/s13054-014-0656-0 (PMC4301454; doi:10.1186/s13054-014-0656-0)
Supplement: Additional file 1 — Target terms for literature search. [file 13054_2014_656_MOESM1_ESM.docx]

**Target terms for literature search**

cardiac surgery, cardiac operation, cardiac operations, heart surgery, heart operation, heart operations, coronary surgery, coronary operation, coronary operations, vascular surgery, vascular operation, vascular operations, valvular surgery, valvular operation, valvular operations, valve surgery, valve operation, valve operations, bypass, myocardial revascularization, valve replacement,

AND

hydroxyethyl starch, hydroxyethyl starches, HES OR 130/0.4, tetrastarch, tetrastarches, Voluven, pentastarch, pentastarches, 200/0.5, hetastarch, hetastarches,

AND

Colloid, colloids, albumin, albumins, gelatin, gelatins, crystalloid, crystalloids, saline, Ringer, Ringer's.
