# Supplementary figures and images for: The impact of hydroxyethyl starches in cardiac surgery: a meta-analysis
Source: Crit Care. 2014 Dec 4;18(6):656. doi: 10.1186/s13054-014-0656-0 (PMC4301454; doi:10.1186/s13054-014-0656-0)

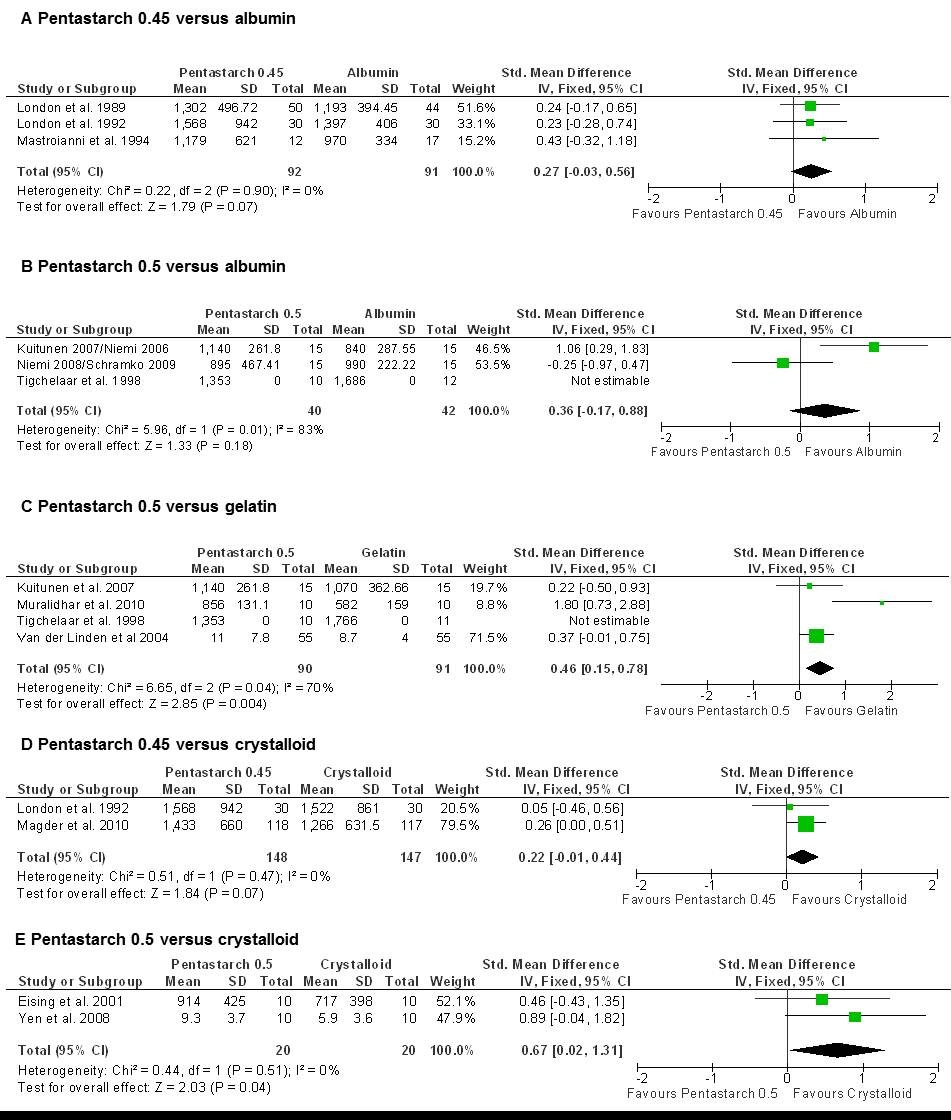

Supplement: Additional file 2 — Blood loss with pentastarch compared to albumin, gelatin or crystalloids. Units of blood loss were millilitres (ml), except for Van der Linden et al. [52], where units were millilitres per kilogram body weight. The standardized mean difference of the mean for the pentastarch groups minus the mean for the albumin, gelatin and crystalloid groups was used as effect size. Fixed-effect models were applied to calculate a common effect estimate using the inverse variance method. Tigchelaar et al. [37] only report mean blood loss without presenting standard deviation (indicated by a ‘0’ in this figure). SD, Standard deviation; Std. mean difference, Standardized mean difference; IV, Inverse variance method; CI, Confidence interval. [file 13054_2014_656_MOESM2_ESM.jpeg]

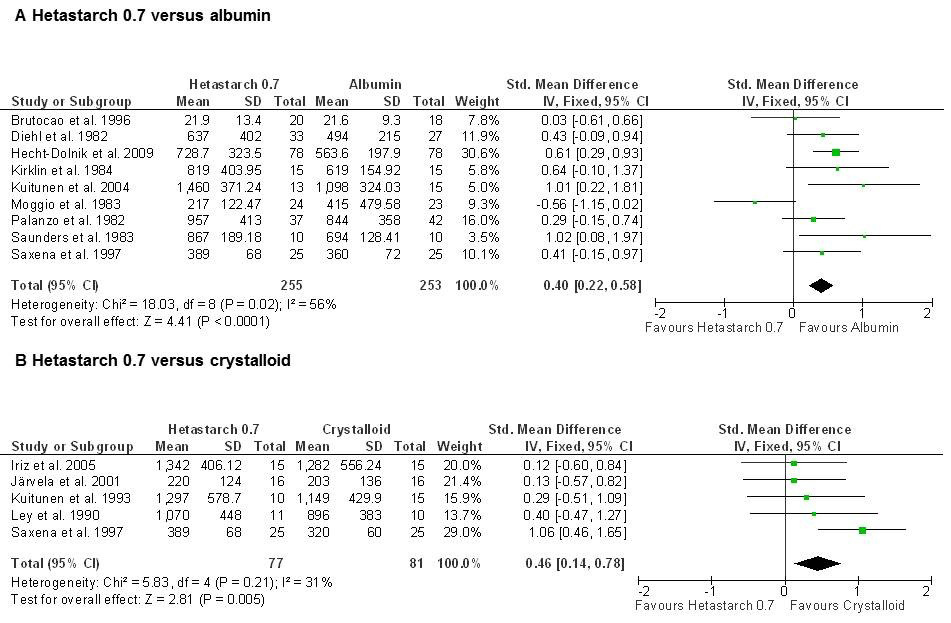

Supplement: Additional file 3 — Blood loss with hetastarch compared to albumin or crystalloids. Units of blood loss were millilitres (ml), except for Brutocao et al. [14], where units were millilitres per kilogram body weight (ml/kg), and Palanzo et al. [41], where no unit was indicated. The standardized mean difference of the mean for the hetastarch groups minus the mean for the albumin and crystalloid groups was used as effect size. Fixed-effects models were applied to calculate a common effect estimate using the inverse variance method. SD, Standard deviation; Std. mean difference, Standardized mean difference; IV, Inverse variance method; CI, Confidence interval. [file 13054_2014_656_MOESM3_ESM.jpeg]

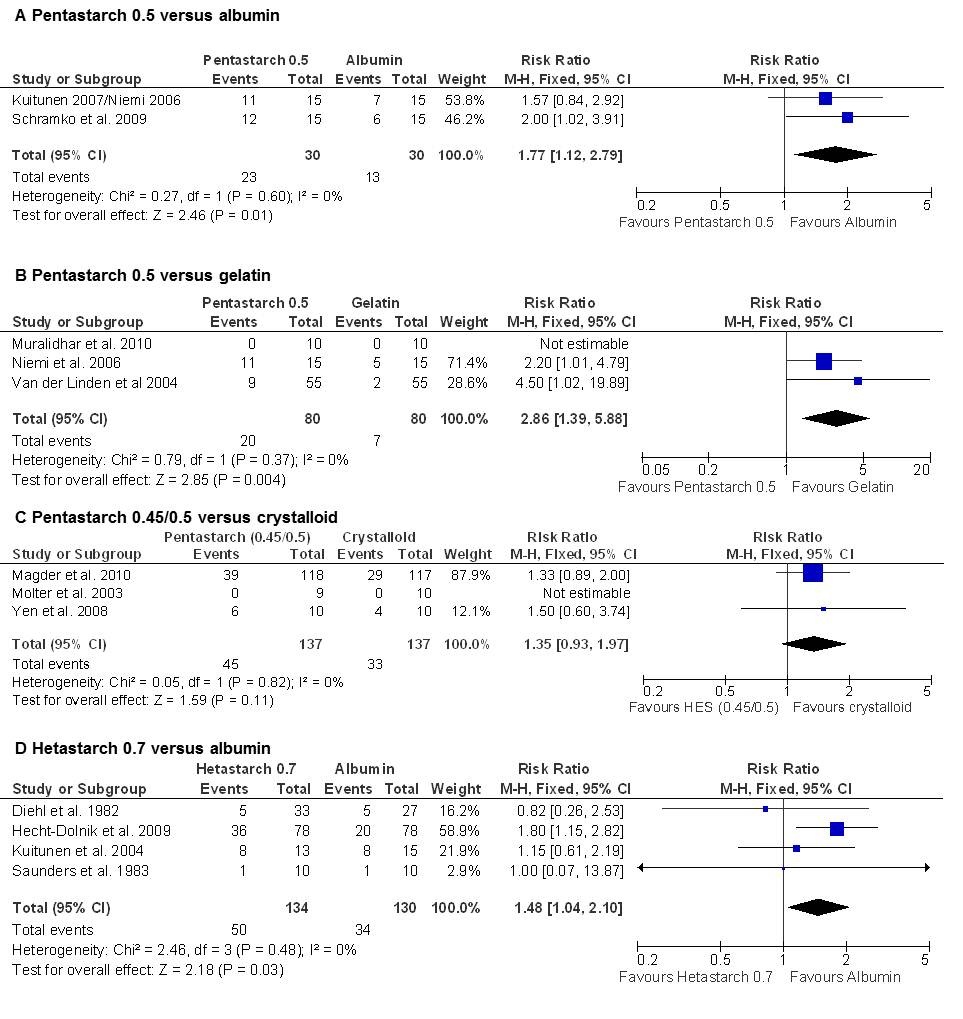

Supplement: Additional file 4 — Transfusion requirements after penta- or hetastarch compared to albumin, gelatin, or crystalloids. The risk ratio was used as effect size (transfusion risk for the penta- or hetastarch groups divided by transfusion risk for the albumin, gelatin and crystalloid groups). Fixed effect models were applied to calculate a common effect estimate using the Mantel-Haenszel approach. M-H, Mantel-Haenszel approach; CI, Confidence interval. [file 13054_2014_656_MOESM4_ESM.jpeg]

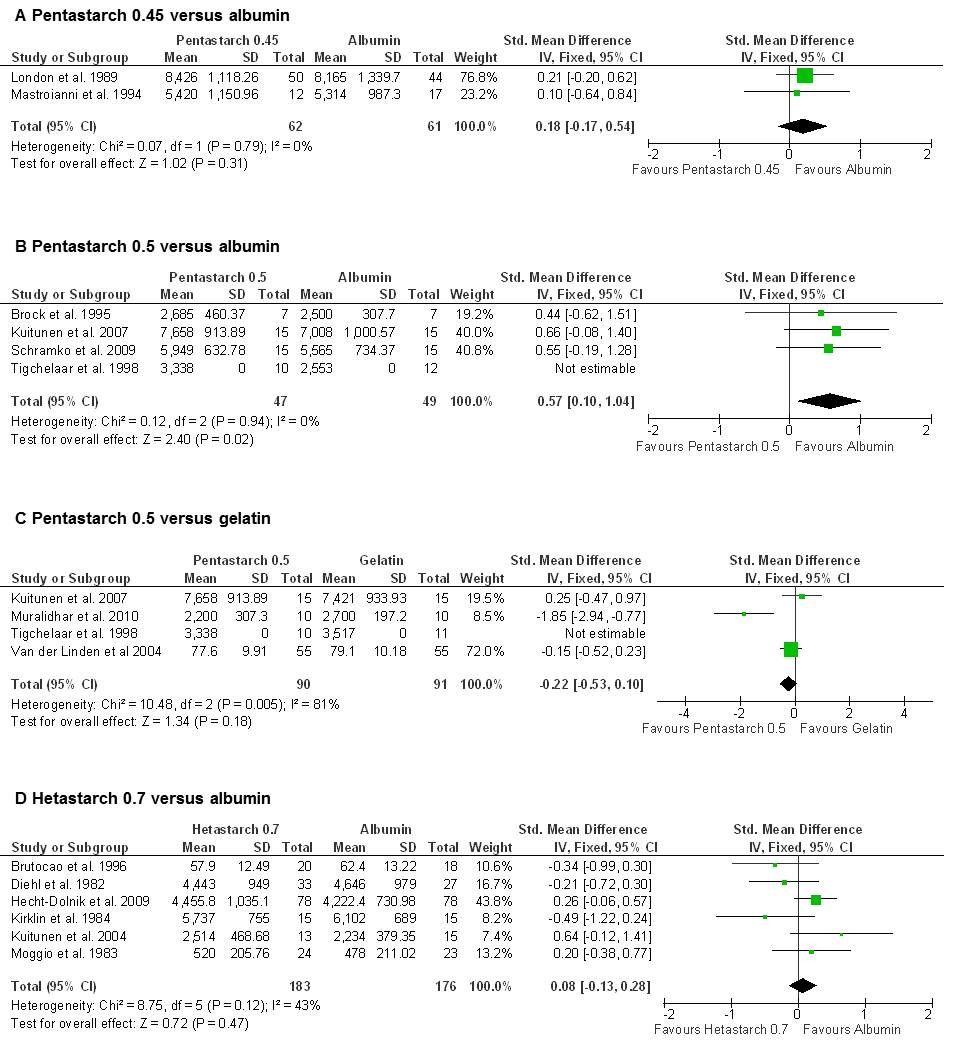

Supplement: Additional file 5 — Length of stay in ICU or hospital after pentastarches compared to albumin and crystalloids. The standardized mean difference of the mean for the pentastarch group minus the mean for the albumin group was used as effect size. A fixed-effects model was applied to calculate a common effect estimate using the inverse variance method. SD, Standard deviation; Std. mean difference, Standardized mean difference; IV, Inverse variance method; CI, Confidence interval. [file 13054_2014_656_MOESM5_ESM.jpeg]

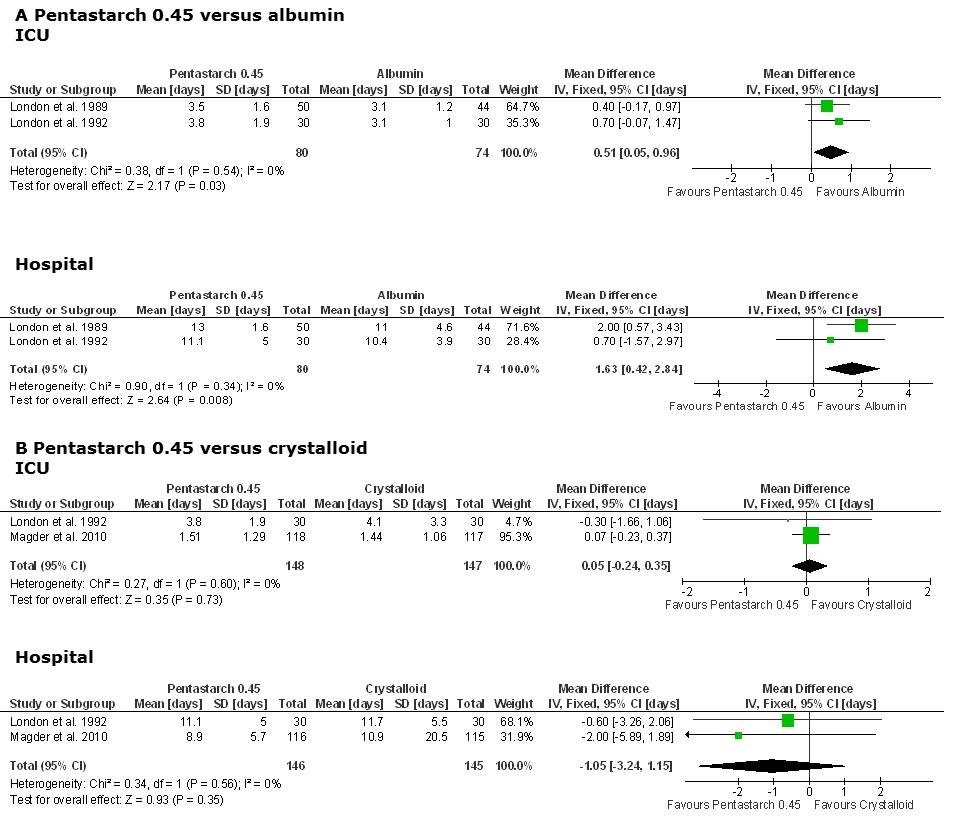

Supplement: Additional file 6 — Length of stay in ICU or hospital after hetastarches compared to albumin and crystalloids. The standardized mean difference of the mean for the pentastarch group minus the mean for the albumin group was used as effect size. A fixed-effect model was applied to calculate a common effect estimate using the inverse variance method. SD, Standard deviation; Std. mean difference, Standardized mean difference; IV, Inverse variance method; CI, Confidence interval. [file 13054_2014_656_MOESM6_ESM.jpeg]
